# Induced biochemical variations in maize parental lines affect the life table and age-specific reproductive potential of *Spodoptera frugiperda* (J.E. Smith)

**DOI:** 10.3389/fpls.2024.1517848

**Published:** 2024-12-09

**Authors:** K. S. Ishwarya Lakshmi, Mukesh K. Dhillon, Ganapati Mukri, K. R. Mahendra, K. V. Gowtham, Aditya K. Tanwar

**Affiliations:** ^1^ Division of Entomology, ICAR-Indian Agricultural Research Institute, New Delhi, India; ^2^ Division of Genetics, ICAR-Indian Agricultural Research Institute, New Delhi, India

**Keywords:** *Spodoptera frugiperda*, maize parental lines, phytochemicals, life table, antibiosis

## Abstract

In recent years, the fall armyworm, *Spodoptera frugiperda* has rapidly emerged as a global invasive pest, challenging the maize production and leading to considerable economic losses. Developing resistant hybrids is essential for sustainable maize cultivation, which requires a comprehensive understanding of resistance traits and the underlying mechanisms in parental lines. To address this need, the present study aimed to identify the sources of resistance, age and stage-specific effects and role of phytochemicals in plant defense against *S. frugiperda* in thirty diverse maize parental lines [17 female (A) and 13 male (R) lines]. The study revealed that the larvae fed on maize A-lines CML 565, AI 501, AI 544 and PDIM 639, and R-lines AI 125, AI 542, AI 155, AI 1100 and PML 105 exhibited a reduced intrinsic (r) and finite rate of increase (λ), and net (R_0_) and gross reproduction rates (GRR); while, increased mean generation time (T) and doubling time (DT). Among these, A-lines CML 565, PDIM 639 and AI 544, and R-lines AI 125, AI 155 and AI 1100 showed higher detrimental effect on reproductive value of *S. frugiperda*. Aforesaid A- and R-lines were also found with greater increase in insect-induced test phytochemicals compared to other lines, accounting for 25.0 to 72.8% variation in the life table parameters, indicating antibiosis effect on *S. frugiperda*. Among the test phytochemicals, tannins, CAT, PAL, TAL and APX inflicted greater effect, indicating their role in induced-biochemical defense against *S. frugiperda.*

## 1 Introduction

The fall armyworm (FAW), *Spodoptera frugiperda* (J.E. Smith), native to Americas and made its first appearance in south India in 2018, infesting maize (Kalleshwaraswamy et al., 2018). It inflicts damage on 353 plant species (Montezano et al., 2018), and poses a major global threat to food security. The larvae of *S. frugiperda* causes severe defoliation of maize plants, resulting in significant reduction of crop yields (Fotso Kuate et al., 2019). The estimated yield losses owing to *S. frugiperda* infestation is around 30% in maize; which however, under favorable conditions, can escalate to as much as 70% (De Groote et al., 2020). Though the timely application of synthetic insecticides effectively manages the *S. frugiperda*, indiscriminate use of insecticides has raised concerns regarding toxicity to humans and the environment (Bolzonella et al., 2019; Rezende-Teixeira et al., 2022). Under such situations, use of resistant cultivars could be the most potential option to manage this pest (AL-Kahtani et al., 2023). However, developing insect-resistant cultivars requires identifying resistance sources within existing germplasm (Arifie et al., 2023). Resistance to insect pests often varies between the male and female parents in crop plants, underscoring the need to assess parental effects (Ni et al., 2024). Male and female parental lines contribute different sets of genes which regulate the defense traits like phenols, leaf toughness and nutrient composition which affect plant resistance to pests. For instance, maternal effects, such as cytoplasmic inheritance, can affect the expression of genes related to defense responses, like secondary metabolites and structural barriers, which are key to deterring insect pests (Botet and Keurentjes, 2020). Therefore, a comprehensive evaluation of the plant traits that enhance insect resistance is essential for overcoming the challenges encountered in insect resistance breeding program.

The most commonly assessed resistance categories against insect pests include antixenosis, antibiosis and tolerance. Several studies have explored these mechanisms across different host plants against *S. frugiperda* (Costa et al., 2019; Correa et al., 2022; Fonsêca et al., 2022; Gual et al., 2023; Nuambote-Yobila et al., 2023; Kuroda et al., 2024). A thorough understanding of the biological and ecological attributes of the insect pest and its interaction with host plant is essential to evaluate the mechanism of resistance in crop plants (Montezano et al., 2018; Wang et al., 2020). Many studies have investigated the biology of *S. frugiperda* on various host plants, that have demonstrated the variations in various biological attributes of *S. frugiperda* across diverse host plants (da Silva et al., 2017; Altaf et al., 2022; Keerthi et al., 2023; Nurkomar et al., 2023; Wang et al., 2023). Life table of an insect is a detailed summary that presents the survival and rate of reproduction considering each developmental stage. It will provide deeper insights into how different host plants influence the development and survival of insect pests (Atlihan et al., 2017), that can be used in screening diverse germplasm to identify least suitable genotypes to the target pest. Since its invasion in India, several traditional life table studies were conducted on *S. frugiperda*, demonstrating various population growth and mortality factors (Ashok et al., 2020; Aralimarad et al., 2024; Sharma et al., 2024). Even though, traditional life tables have long been used in entomological studies, they have notable limitations like exclusion of male populations, various developmental stages, and individual variations within the population that affect the accuracy of describing insect population characteristics (Birch, 1948). Recognizing these limitations, researchers have shifted to the age-stage, two-sex life table, which incorporates both sexes and provides a more comprehensive view of population characteristics (Chi et al., 2023). This advanced framework has been widely applied to study various aspects of S. frugiperda biology and behaviour (He et al., 2021a; Xie et al., 2021; Chen et al., 2022; Zhang et al., 2023). Despite the progress made in understanding the biology of *S. frugiperda*, there has been limited exploration of age-stage, two-sex based life table analyses for screening of diverse maize parental lines in Indian conditions.

The development and reproduction of an insect depend on the biochemical composition (Liu et al., 2022; Nelly et al., 2023) of host plant, that can be differed considerably among various host plants. Hence, evaluation of biological traits of insects along with the plants biochemical components is crucial for assessing resistance to insect pests (Sau and Dhillon, 2022). The biochemical defense mechanisms in crop cultivars are regulated by constitutive and/or induced compounds. Plant secondary metabolites such as phenols and tannins are considered antinutritional as they are not involved in plant growth rather impart plant defense against stresses, and at the same time makes less preferred host for the herbivores (War et al., 2012). Insect herbivory triggers plant to produce various secondary metabolites, antinutritional compounds and toxic proteins, that disrupt the biological functions of insects (Smith and Clement, 2012). Reactive oxygen species (ROS) are produced in response to biotic stress, which functions as secondary messenger to trigger defense reaction in host plants (Asada, 2006). In this process, various naturally occurring plant antioxidants and enzymes, such as ascorbate oxidase (AO), ascorbate peroxidase (APX), catalase (CAT), phenylalanine ammonia lyase (PAL) and tyrosine ammonia lyase (TAL) are increased that play key roles in detoxifying ROS and maintaining redox balance (Gill and Tuteja, 2010; Huang et al., 2019). Secondary metabolites in plants, such as tannins and phenols can also trigger additional defenses (Isah, 2019; Singh et al., 2021). Over the past few decades, research on plant-induced resistance to various stresses has significantly advanced becoming a crucial area for understanding plant-herbivore interactions (War et al., 2012). Although, several earlier studies have explored sources of resistance to *S. frugiperda* (Wiseman et al., 1967; Williams and Davis, 2002), the specific underlying resistance mechanisms in diverse parental maize lines remains unclear. Additionally, there is limited knowledge on the age- and stage-specific effect of maize parental lines and fate of *S. frugiperda*-induced antioxidant defense biochemicals on its life table parameters. Hence, the current study was designed to decipher the effect of test maize parental lines on the life table parameters, regulation of certain plant defensive biochemicals due to *S. frugiperda*-damage, and association of induced levels of these biochemicals with the growth and reproduction of *S. frugiperda*. The studies will help in identifying the fall armyworm-resistant maize parental lines and associated defense mechanisms, having implications for breeding program to develop maize hybrids conferring resistance to *S. frugiperda*.

## 2 Materials and methods

### 2.1 Plant material and crop raising

Thirty diverse maize lines [17 A-lines (female) and 13 R-lines (male)] including resistant (CML 442) and susceptible (UM 1210) checks were used in the current study. The majority of these maize lines are potential parent lines that can be used in developing medium- to long- duration hybrids. The lines were chosen because of their advantageous inbred qualities in both the females (greater cob attributes) and males (strong pollen donors) parents. They also show moderate to high general combining ability, which increases the possibility of passing on desired qualities like resistance. The parental maize lines were grown in 4-row plots of 3m length at 60 x 30 cm spacing in the experimental fields of Division of Entomology, IARI, New Delhi during *Kharif* 2022 and 2023. The experiment was conducted in a completely randomized block design with three replications. All recommended agronomic practices, including thinning, weeding, irrigation and fertigation were followed, except for insecticide application to grow these maize parental lines.

### 2.2 Developmental biology of *S. frugiperda* across diverse maize parental lines

The *S. frugiperda* culture for this study was reared on an artificial diet (Gopalakrishnan and Kalia, 2022) under controlled conditions of 27 ± 2°C and 65-75% relative humidity and 12h light:12h dark photoperiod, at the Division of Entomology, ICAR-Indian Agricultural Research Institute (IARI), New Delhi. The biology of *S. frugiperda* on diverse parental maize lines was investigated using seedlings of field-grown maize lines. Twenty-five neonates were individually placed in 5.5 cm Petri plate and fed on the leaves of 25 days old seedlings of test maize lines. There were three replications for each maize line. Once the larvae reached the second instar, they were fed on the stems of 30 days old plants of respective maize lines, which were replaced every alternate day, continuing until pupation. On the second day of pupation, male and female pupae were separated, individually weighed and placed in 5.5cm Petri plate. Observations were collected on the number of larval instars, the duration of each instar (days), survival rates (%) at each stage, pupal duration (days) and survival rates (%) at adult emergence. After adult emergence, five male and five female adults were placed in an oviposition jar and there were three replications for each treatment. The honey solution (5%) and egg cards for egg laying were placed in each oviposition cage. Observations were recorded on daily fecundity (eggs per female), pre-oviposition period (days), oviposition period (days), post-oviposition period and egg incubation period (days) for each treatment.

### 2.3 Analysis of the age-stage, two-sex life table

The life table analysis of *S. frugiperda* reared on maize parental lines was performed using the TWO-SEX-MS Chart program, which is based on the age-stage, two-sex life table theory (Chi and Liu, 1985; Chi, 1988, 2023; Huang and Chi, 2013) and the methodologies established by Tuan et al. (2014). The data obtained from the biology study was used for this purpose. The life table analysis focused on key population parameters such as the intrinsic rate of increase (r), finite rate of increase (λ), net reproductive rate (R_0_), gross reproduction rate (GRR), mean generation time (T) and doubling time (DT), along with reproductive value curves (*v_xj_*) of *S. frugiperda*. They were determined by using the following formulae

- The intrinsic rate of increase (r): The growth rate of a population when it reaches a stable age-stage distribution. At, this point, the population size will increase at a rate of e^r^ per time unit and determined using the formula: $\;\sum_{x=0}^{\infty }e^{-r\left(x+1\right)}l_{x}m_{x}=1$ Where ‘e’ is the base of natural logarithm (2.71828), *‘l_x_*’ is the age-specific survival rate and ‘*m_x_*’ is the female age-specific fecundity.
- The finite rate of increase (λ): It is the population growth rate as time approaches infinity and population reaches the stable age-stage distribution. At, this stage, the population size will increase at the rate of λ per time unit: $\;\;\text{λ}=e^{r}$ .
- The net reproductive rate (R0): the total number of offsprings produced by an individual over its lifetime: $R_{0}=\sum_{x=0}^{\infty }l_{x}m_{x}$ .
- The gross reproduction rate (GRR): The gross reproduction rate is calculated by adding up the age-specific fertility rates for female live births: $\text{GRR}{=}^{\sum }m_{x}$ .
- The mean generation time (T): The average interval between the birth of an individual and the birth of its offsprings: $\text{T}=\frac{ln\;R_{0}}{r}$ .
- The doubling time (DT): The time it takes for a population to double in size/value: $\text{DT}=\frac{ln\;2}{r}$ .
- Age-stage-specific reproductive value (*v_xj_*): It represents the contribution of an individual of age *x* and stage *j* to the future population (Tuan et al., 2014): $v_{xj}=\frac{e^{r\left(x+1\right)}}{S_{xj}}\sum_{i=x}^{\infty }e^{-r\left(i+1\right)}\sum_{y=j}^{m}S{'}_{iy}f_{iy}$ .

### 2.4 Raising, collection and processing of maize seedling samples for estimation of different biochemical constituents

Maize seedlings were raised in plastic pots (26 cm height and 26 cm diameter) under shade net (50 m^2^ area covered with a nylon net of 4 mm mesh size) with each maize line sown in four pots (ten seedlings per pot). On the 25^th^ day, two pots were infested with three-second instar *S. frugiperda* larvae while two were kept uninoculated as control. After 48 hours of infestation, healthy and damaged seedlings were collected separately and procced immediately for biochemical analysis. Two grams of stem tissue from each line were ground in liquid nitrogen, mixed with 10 ml of 50 mM phosphate buffer (pH 7.8), and centrifuged at 12,000 rpm for 20 minutes at 4°C. The supernatant was collected in 2.5 ml Eppendorf tubes and stored at -20°C for further biochemical analysis.

### 2.5 Estimation of constitutive and induced levels of biochemicals in test maize lines

The nutritional compounds, such as total sugar and total soluble proteins, were analyzed in the test maize parental lines. Total sugar content was analyzed using the concentrated sulfuric acid method (Dubois et al., 1956) using glucose as the standard, while total soluble protein was quantified using the Bradford method (Bradford, 1976) with bovine serum albumin (BSA) as the standard. Anti-nutritional compounds, such as total phenol and tannin were determined following the methods of Singleton and Rossi (1965) and Amorim et al. (2008), respectively. Total antioxidant and ferric ion-reducing antioxidant power (FRAP) contents were determined using protocols by Prieto et al. (1999) and Benzie and Strain (1999), respectively. The values of all the biochemicals were expressed as mg/g of plant tissue. The activity of antioxidant enzymes in maize samples *viz*., ascorbate oxidase (AO), ascorbate peroxidase (APX), tyrosine ammonia lyase (TAL), phenylalanine ammonia lyase (PAL) and catalase were estimated using the methods given by Diallinas et al. (1997); Ali et al. (2005); Beaudoin-Eagan and Thorpe (1985); Fritz et al. (1976) and Aebi (1984), respectively, and expressed as U/ml of enzyme extract, where U denotes μmol/min.

The change in the levels of the above-mentioned phytochemicals in the seedlings of test parental inbred lines due to *S. frugiperda* damage were calculated using the following formula:


$$\text{Change in biochemical content}\left(\%\right)=\frac{\text{Quantity in damaged seedlings }-\text{Quantity in healthy seedlings }}{\text{Quantity in healthy seedlings }}\times 100$$

### 2.6 Statistical analysis

The life table on *S. frugiperda* and the insect damage-induced biochemicals of maize parental lines were subjected to a one-way analysis of variance using R Studio analysis software^®^ version 4.4.1. The significance of differences was assessed using the *F*-test, and treatment averages were compared using the least significant difference (LSD) method at a significance level of *P =* 0.05. Further, the Pearson correlation and regression analysis were performed to decipher the association between the induced levels of plant biochemicals and life table parameters of *S. frugiperda* using the same software. Based on the results of life table analysis, eight of the best-performing maize lines (three A-lines and five R-lines) were selected for estimating the reproductive value, and compared with resistant and susceptible checks. The reproductive values (*v_xj_*) were calculated using the TWO-SEX-MS Chart program, and graphs were created using Microsoft Excel 2019.

## 3 Results

### 3.1 Life table parameters of *S. frugiperda* on various maize lines

Population growth parameters of *S. frugiperda* developed on diverse male (R-lines) and female (A-lines) maize lines varied significantly (**Table 1**). The intrinsic rate of increase (*F_29,60_ = 2.82; P<0.001*) of *S. frugiperda* varied between 0.10 to 0.15 per day with lower rates on A-lines CML 565, PDIM 639, AI 544, DDM 2309-O and AI 196, and R- lines AI 125, AI 155, AI 1100 and PML 105 than the susceptible check UMI 1210. However, the finite rate of increase (*F_29,60_ = 9.93; P=0.001*) was ranged from 1.01 to 1.20 per day and significantly lower on A-lines CML 565, and R-lines AI 125 (**Table 1**). Further, the net reproductive rate (*F_29,60_ = 19.78; P<0.001*) and the gross reproduction rate (*F_29,60_ = 21.31; P<0.001*) ranged from 44.76 to 96.32 and 102.61 to 220.21 eggs per female, respectively with lower rates on A-lines CML 565, AI 544 and PDIM 639, and R-lines AI 125, AI 155, AI 1100 and AI 542 than susceptible check UMI 1210 (**Table 1**). Furthermore, the mean generation time (*F_29,60_ = 1.97; P=0.014*) and doubling time of *S. frugiperda* (*F_29,60_ = 4.18; P<0.001*) ranged from 29.20 to 38.53 and 4.57 to 6.93 days, respectively. The mean generation time was significantly higher on A-lines CML 565, AI 196, AI 142[R], DDM 2309-O and AI 501, and R-lines AI 125, AI 155, AI 545, AI 1100 and AI 542 than the susceptible check UMI 1210 but was similar to that of the other lines. Similarly, as compared to susceptible check UMI 1210, the doubling time was significantly higher on A-lines CML 565, AI 544 and AI 196, and R-lines AI 125, AI 542, AI 155 and PML 105 which however, was on par with resistant check CML 442 (**Table 1**).

**Table 1:** Life table parameters of *Spodoptera frugiperda* on various male and female maize lines.

Maize lines	r (d^-1^)	λ (d^-1^)	Ro (offsprings/female)	GRR (offsprings/female)	T (days)	DT (day)
A-lines (female lines)						
AI 142[R]	0.13 ± 0.01	1.14 ± 0.01	82.56 ± 3.79	176.51 ± 6.10	36.38 ± 0.40	5.38 ± 0.33
AI 178	0.13 ± 0.01	1.13 ± 0.01	85.92 ± 3.54	220.21 ± 8.23	34.17 ± 1.45	5.59 ± 0.13
AI 544	0.11 ± 0.01	1.11 ± 0.01	47.28 ± 2.60	128.44 ± 5.47	35.69 ± 0.74	6.93 ± 0.44
AI 546	0.13 ± 0.01	1.20 ± 0.00	84.20 ± 1.23	189.93 ± 6.56	34.05 ± 1.63	5.32 ± 0.37
AI 196	0.12 ± 0.01	1.10 ± 0.02	70.20 ± 3.55	136.14 ± 9.27	37.10 ± 0.54	6.57 ± 0.46
AI 1116	0.12 ± 0.01	1.13 ± 0.02	79.36 ± 5.52	156.25 ± 8.57	35.39 ± 0.94	5.61 ± 0.34
AI 501	0.13 ± 0.01	1.14 ± 0.02	70.92 ± 2.65	137.60 ± 7.23	35.94 ± 1.50	5.82 ± 0.35
AI 518	0.13 ± 0.00	1.14 ± 0.01	76.52 ± 4.89	213.67 ± 13.85	34.10 ± 0.70	5.45 ± 0.26
AI 540	0.12 ± 0.01	1.13 ± 0.01	84.28 ± 3.47	169.53 ± 9.06	35.55 ± 0.77	5.56 ± 0.31
DMS 4B	0.13 ± 0.00	1.13 ± 0.02	81.40 ± 2.66	147.12 ± 8.56	35.05 ± 2.41	5.36 ± 0.23
CML 565	0.10 ± 0.00	1.01 ± 0.01	44.76 ± 1.23	115.19 ± 4.69	38.53 ± 0.49	6.51 ± 0.20
PDM 77-A	0.12 ± 0.00	1.13 ± 0.02	76.00 ± 2.81	189.11 ± 4.30	32.79 ± 1.97	5.65 ± 0.14
PDIM 639	0.10 ± 0.01	1.13 ± 0.01	50.16 ± 2.14	136.64 ± 9.12	35.69 ± 0.56	5.02 ± 0.16
PDM 6555	0.14 ± 0.01	1.15 ± 0.01	93.12 ± 5.02	164.77 ± 8.96	34.44 ± 1.35	5.29 ± 0.17
C 70	0.13 ± 0.01	1.13 ± 0.01	92.08 ± 6.10	161.73 ± 8.11	35.32 ± 0.47	5.41 ± 0.24
DDM 2309-O	0.12 ± 0.01	1.15 ± 0.01	73.52 ± 3.79	140.23 ± 4.78	35.91 ± 1.29	5.79 ± 0.32
C 11	0.13 ± 0.00	1.14 ± 0.01	96.32 ± 5.01	195.12 ± 4.71	32.52 ± 0.32	5.52 ± 0.10
R-lines (male lines)						
AI 117	0.13 ± 0.01	1.14 ± 0.02	94.16 ± 5.79	189.08 ± 2.99	35.01 ± 1.84	5.34 ± 0.13
AI 125	0.11 ± 0.00	1.02 ± 0.01	48.68 ± 1.59	102.61 ± 5.34	37.21 ± 0.72	6.39 ± 0.10
AI 541	0.13 ± 0.01	1.11 ± 0.01	68.68 ± 2.50	122.61 ± 1.28	33.64 ± 0.57	5.35 ± 0.24
AI 155	0.11 ± 0.01	1.11 ± 0.01	49.45 ± 0.96	123.75 ± 5.09	37.00 ± 1.82	6.36 ± 0.21
AI 545	0.12 ± 0.00	1.13 ± 0.02	80.60 ± 4.73	185.47 ± 5.26	35.98 ± 1.43	5.68 ± 0.22
AI 1100	0.11 ± 0.00	1.12 ± 0.01	54.96 ± 3.04	125.18 ± 5.75	35.63 ± 0.46	5.51 ± 0.07
AI 542	0.12 ± 0.01	1.12 ± 0.02	56.20 ± 3.96	132.00 ± 3.69	35.29 ± 0.56	6.37 ± 0.14
PML 105	0.11 ± 0.01	1.12 ± 0.02	68.56 ± 1.82	135.68 ± 2.50	35.06 ± 0.43	6.10 ± 0.40
AI 525	0.13 ± 0.00	1.13 ± 0.01	77.04 ± 4.37	152.52 ± 7.65	33.62 ± 0.55	5.59 ± 0.29
PDM 24-1	0.11 ± 0.01	1.15 ± 0.01	93.68 ± 3.50	188.87 ± 3.22	35.26 ± 1.45	5.37 ± 0.19
PDM 4061	0.13 ± 0.01	1.14 ± 0.01	83.72 ± 2.81	174.36 ± 6.85	34.42 ± 1.97	5.25 ± 0.16
CML 442 (R)	0.11 ± 0.01	1.13 ± 0.01	57.56 ± 3.46	162.40 ± 6.99	36.70 ± 1.54	6.28 ± 0.13
UMI 1210 (S)	0.15 ± 0.01	1.20 ± 0.00	94.96 ± 1.75	210.73 ± 6.08	29.20 ± 1.59	4.57 ± 0.14
F-probability	<0.001	0.001	<0.001	<0.001	0.014	<0.001
LSD (*P* = 0.05)	0.02	0.03	10.26	19.36	3.49	0.72

The values in the table represent Mean ± Standard error; LSD, Least significant differences; R, Resistant check; S, Susceptible check; r, The intrinsic rate of increase; λ, The finite rate of increase; Ro, The net reproductive rate; GRR, The gross reproduction rate; T, The mean generation time; DT, The doubling time.

### 3.2 Reproductive value (*v_xj_*) of each age-stage group of *S. frugiperda* on selected maize lines

**Figure 1:**
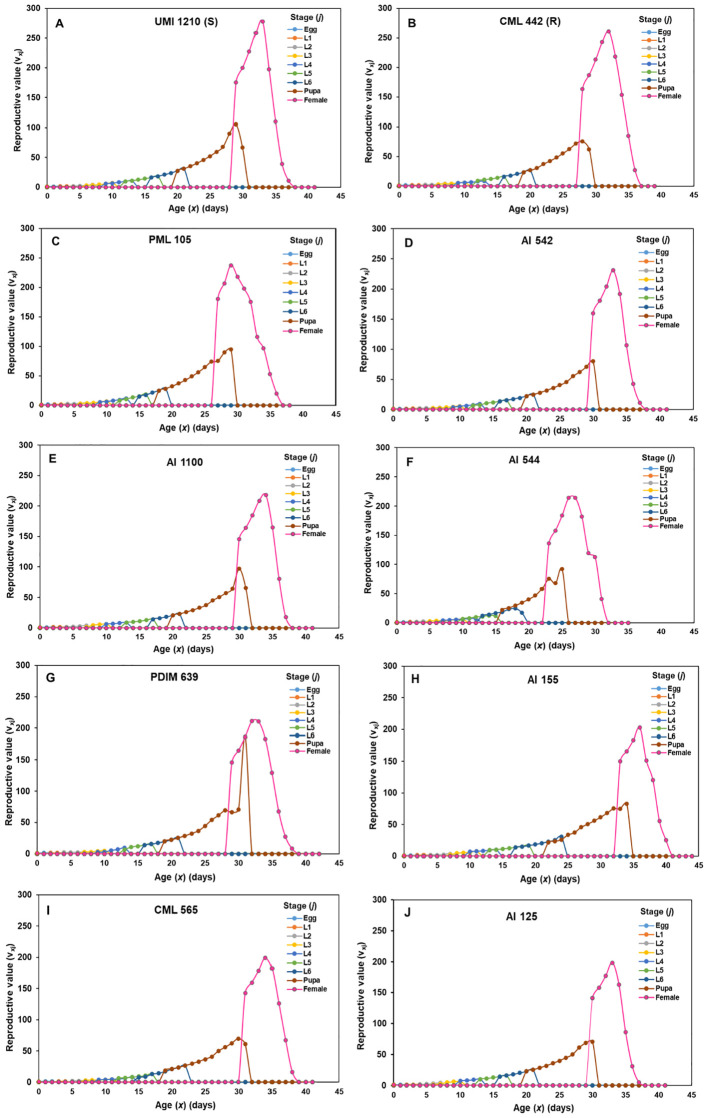
The age-stage reproductive value (*v_xj_*) of *S. frugiperda* on ten diverse parental maize lines **(A–J)**. [The *v_xj_* on susceptible check UMI 1210 **(A)**, resistant check CML 442 **(B)**, PML 105 **(C)**, AI 542 **(D)**, AI 1100 **(E)**, AI 544 **(F)**, PDIM 639 **(G)** and AI 155 **(H)**, CML 565 **(I)** and AI 125 **(J)**].

Based on the results of life table analysis, eight best-performing maize lines (three A-lines and five R-lines) were selected for constructing reproductive value (*v_xj_*) graphs and they were compared with *v_xj_* of resistant and susceptible checks. The reproductive value of *S. frugiperda* initially increased, but then declined to zero as the development stage progressed (**Figures 1A–J**). Each maize line *v_xj_* curve exhibited a significant rise after adult emergence, reaching a peak, often coinciding with the pupal curve. The female adults reached their highest reproductive peak at the 33^rd^ day on susceptible check UMI 1210 (*v_33,9_* = 277.88), followed by resistant check CML 442 (*v_32,9_* =260.74), PML 105 (*v_29,9_* = 237.44), AI 542 (*v_33,9_* = 230.88), AI 1100 (*v_34,9_* = 218.32), AI 544 (*v_27,9_* = 214.27), PDIM 639 (*v_32,9_* = 211.01), AI 155 (*v_36,9_* = 202.86), CML 565 (*v_34,9_* = 198.87), AI 125 (*v_33,9_* = 198.41), (**Figures 1A–J**). These maize lines showed a higher detrimental effect on *v_xj_* of *S. frugiperda* as compared to other lines including the resistant check, CML 442.

### 3.3 Change in the levels of nutritional and antinutritional constituents in the *S. frugiperda* damaged over healthy maize seedlings

Total sugar and protein levels were increased across the seedlings of maize parental lines damaged by the *S. frugiperda* and ranged from 9.1 to 52.8 and 14.2 to 53.9%, respectively with significant variation for both sugars (*F_29,60_ = 304.22; P<0.001*) and proteins (*F_29,60_ = 300.19; P<0.001*) (**Table 2**). The increment of total sugar content was lesser in A-line C 70, DMS 4B, CML 565 and AI 501 and R-line AI 542, AI 125, PDM 4061, AI 117 and AI 110 when compared to both the checks. Further, the upsurge of total protein content was significantly lower in A-lines CML 565, AI 501, DMS 4B and PDM 77-A, and R-lines AI 125, AI 525, AI 1100 and AI 542 compared to all other test lines (**Table 2**). Total phenol and tannin content was increased in the seedlings of *S. frugiperda* damaged maize line and varied from 11.5 to 61.8% and 6.4 to 31.1%, respectively. The per cent change in total phenol content (*F_29,60_* = 166.98; *P<0.001*) were significantly higher in A-lines AI 178, AI 501, CML 565 and AI 1116, and R-lines AI 542, AI 1100, AI 125 and AI 155 as compared to other lines including both the checks. Further, the per cent change in total tannins (*F_29,60_* = 18.70; *P<0.001*) was significantly higher on A-lines CML 565, AI 501, AI 1116 and AI 544, and R-lines AI 1100, AI 542, AI 125 and resistant check CML 442 (**Table 2**). The per cent increase of total antioxidant content in the *S. frugiperda* damaged seedlings varied significantly among the maize genotypes (*F_29,60_* = 16.99; *P<0.001*) and ranged from 12.9 to 37.0%. This increase was highest in the A-lines AI 501, CML 565 and PDIM 639, and R-lines AI 125 and AI 1100 as compared to other test maize lines. Similarly, the per cent increase of ferric ion-reducing antioxidant power (FRAP) in the *S. frugiperda* damaged seedlings varied significantly among the maize genotypes (*F_29,60_* = 47.02; *P<0.001*) and ranged from 21.0 to 59.4%. The increase was significantly greater in the A-lines CML 565 and AI 501, and R-lines AI 125 and AI 542 as compared to all other test lines including both the checks (**Table 2**).

**Table 2:** Change in the levels of nutritional and antinutritional constituents in the *Spodoptera frugiperda* damaged over healthy maize seedlings.

Maize lines	Total sugars (%)	Total proteins (%)	Total phenols (%)	Total tannins (%)	Total antioxidants (%)	FRAP (%)
A-lines (female lines)						
AI 142 [R]	48.3 ± 0.5	42.8 ± 0.9	11.5 ± 0.1	6.4 ± 0.2	15.1 ± 1.6	30.0 ± 1.9
AI 178	23.3 ± 1.0	51.5 ± 0.6	34.0 ± 0.2	19.3 ± 1.3	30.8 ± 0.5	33.6 ± 1.1
AI 544	52.8 ± 0.2	38.0 ± 0.2	17.3 ± 0.6	21.9 ± 1.2	22.5 ± 0.9	29.1 ± 1.3
AI 546	25.1 ± 0.9	53.6 ± 0.7	21.8 ± 0.9	18.4 ± 0.7	25.7 ± 1.7	33.2 ± 1.6
AI 196	23.8 ± 1.0	34.3 ± 0.3	23.7 ± 0.9	18.2 ± 1.2	23.5 ± 1.5	27.7 ± 1.2
AI 1116	32.9 ± 0.5	32.8 ± 0.7	61.8 ± 0.8	24.5 ± 1.6	25.7 ± 2.1	28.5 ± 0.6
AI 501	22.9 ± 0.5	26.0 ± 0.1	34.6 ± 1.0	25.8 ± 1.6	35.3 ± 0.2	41.2 ± 0.6
AI 518	40.3 ± 0.4	46.7 ± 1.6	14.3 ± 0.0	9.5 ± 0.6	19.9 ± 1.0	21.5 ± 0.9
AI 540	31.1 ± 0.2	37.7 ± 0.5	18.3 ± 0.4	20.5 ± 0.7	24.5 ± 1.1	26.7 ± 1.2
DMS 4B	21.1 ± 0.3	26.3 ± 0.6	31.6 ± 0.6	18.6 ± 1.5	22.4 ± 0.7	36.8 ± 2.0
CML 565	21.8 ± 0.2	18.5 ± 0.5	44.2 ± 0.7	31.1 ± 0.2	33.5 ± 1.8	40.8 ± 1.2
PDM 77-A	27.8 ± 0.4	26.9 ± 0.7	30.5 ± 0.3	19.1 ± 0.6	20.9 ± 1.4	29.5 ± 1.4
PDIM 639	34.8 ± 1.5	30.3 ± 0.7	20.3 ± 0.8	19.7 ± 0.9	31.0 ± 0.9	28.3 ± 0.2
PDM 6555	36.4 ± 0.3	29.9 ± 0.7	33.0 ± 0.6	20.7 ± 2.5	24.3 ± 1.4	22.5 ± 0.8
C 70	16.6 ± 0.2	51.4 ± 0.6	26.6 ± 0.9	17.5 ± 0.8	25.6 ± 1.3	30.7 ± 1.0
DDM 2309-O	34.5 ± 0.4	34.9 ± 0.9	17.4 ± 0.4	16.6 ± 2.8	24.1 ± 1.6	29.0 ± 0.5
C 11	30.4 ± 0.6	39.1 ± 0.4	22.4 ± 1.3	21.7 ± 0.7	22.6 ± 0.3	34.2 ± 2.8
R-lines (male lines)						
AI 117	24.2 ± 0.6	37.5 ± 0.4	25.4 ± 0.6	19.3 ± 1.1	24.7 ± 0.7	33.1 ± 1.3
AI 125	15.4 ± 0.3	14.2 ± 0.4	36.2 ± 2.0	22.1 ± 0.4	37.0 ± 0.8	59.4 ± 0.9
AI 541	32.3 ± 0.7	45.9 ± 0.4	15.4 ± 0.6	9.9 ± 0.4	12.9 ± 0.6	32.2 ± 0.9
AI 155	26.3 ± 0.1	51.9 ± 0.5	32.1 ± 0.7	20.0 ± 0.9	20.7 ± 0.9	37.7 ± 0.6
AI 545	45.0 ± 0.6	53.9 ± 0.8	14.9 ± 1.0	11.6 ± 0.2	20.3 ± 3.2	21.6 ± 1.3
AI 1100	24.2 ± 0.7	22.2 ± 0.2	37.4 ± 0.7	26.6 ± 1.7	32.2 ± 0.4	47.3 ± 1.4
AI 542	9.1 ± 0.0	24.3 ± 0.4	50.8 ± 1.3	24.6 ± 1.0	30.7 ± 0.2	51.6 ± 0.3
PML 105	34.3 ± 0.6	28.3 ± 0.8	27.0 ± 1.1	20.8 ± 1.9	25.9 ± 1.0	29.6 ± 1.3
AI 525	27.5 ± 0.1	18.2 ± 0.2	19.4 ± 0.5	20.2 ± 0.9	21.3 ± 0.3	36.8 ± 0.6
PDM 24-1	27.7 ± 0.6	40.9 ± 0.7	20.7 ± 0.6	16.8 ± 1.2	29.4 ± 1.1	37.8 ± 1.8
PDM 4061	22.1 ± 0.5	43.9 ± 1.0	25.6 ± 1.9	20.6 ± 1.2	27.8 ± 2.3	28.9 ± 1.5
CML 442 (R)	34.2 ± 0.5	27.0 ± 0.4	25.6 ± 0.8	21.1 ± 1.4	29.0 ± 2.2	31.2 ± 1.3
UMI 1210 (S)	52.5 ± 0.9	36.1 ± 0.5	18.5 ± 0.5	10.9 ± 0.3	28.6 ± 0.5	21.0 ± 0.4
F-probability	<0.001	<0.001	<0.001	<0.001	<0.001	<0.001
LSD (*P* = 0.05)	1.69	1.84	2.48	3.49	3.81	3.58

The values in the table represent Mean ± Standard error. FRAP, Ferric ion-reducing antioxidant power; LSD, Least significant differences; R, Resistant check; S, Susceptible check.

### 3.4 Change in the various defense enzymes in the *Spodoptera frugiperda* damaged over healthy maize seedlings

The percentage enhancement in the activity of ascorbate oxidase in the *S. frugiperda* induced conditions varied significantly (*F_29,60_ = 12.91; P<0.001*) and ranged from 9.9 to 28.6% with significantly greater increment in A-lines CML 565, C 70, AI 501 and PDM 6555, and R-lines AI 125, AI 525 and AI 1100 than all other test lines including both the checks (**Table 3**). Further, the per cent increase in the activity of ascorbate peroxidase also differed significantly (*F_29,60_ = 12.71; P<0.001*), with greater increment in A-lines AI 501, CML 565 and AI 1116, and R-lines AI 542, AI 125, AI 1100 and AI 155, than all other lines including both the checks (**Table 3**). Furthermore, the changeover of catalase activity (*F_29,60_ = 13.69; P<0.001*) was highest in A-line CML 565 followed by AI 501 and AI 196, and R-lines AI 125, AI 1100, AI 542 and including resistant check CML 442 as compared to remaining maize lines (**Table 3**). Similarly, the per cent increase in the activity of phenylalanine ammonia lyase in the *S. frugiperda induced* conditions also varied significantly (*F_29,60_* = 13.04; *P<0.001*) and ranged from 15.3 to 36.3%, in A-lines CML 565 and AI 501, and R-lines AI 125 and AI 1100 having significantly greater induced levels than all other test lines including both the checks (**Table 3**). The per cent increase in the activity of tyrosine ammonia lyase in the *S. frugiperda* damaged seedlings varied significantly among each other (*F_29,60_* = 4.95; *P<0.001*). This increase was significantly higher in A-lines CML 565, AI 1116 and DDM 2309-O, and R-lines AI 542, AI 1100, AI 125 which were on par with resistant check CML 442 (**Table 3**).

**Table 3:** Change in the activity of enzymatic components in the *Spodoptera frugiperda* damaged over healthy maize seedlings.

Maize lines	Ascorbate oxidase (%)	Ascorbate peroxidase (%)	Catalase (%)	Phenylalanine ammonia lyase (%)	Tyrosine ammonia lyase (%)
A-lines					
AI 142 [R]	15.3 ± 2.3	15.1 ± 1.6	20.2 ± 1.2	18.9 ± 0.5	10.3 ± 0.4
AI 178	21.5 ± 0.0	21.0 ± 2.3	24.9 ± 2.3	22.5 ± 1.7	15.7 ± 2.9
AI 544	14.0± 0.8	14.2 ± 1.2	23.4 ± 0.8	23.9 ± 0.1	17.1 ± 1.6
AI 546	16.2 ± 1.1	15.8 ± 1.3	19.9 ± 1.9	23.7 ± 1.9	18.1 ± 2.0
AI 196	14.3 ± 0.8	17.2 ± 2.3	27.5 ± 2.0	21.0 ± 1.4	15.8 ± 1.9
AI 1116	15.7 ± 0.8	22.9 ± 1.5	25.6 ± 1.7	21.1 ± 1.5	20.8 ± 2.4
AI 501	23.4 ± 2.4	26.2 ± 0.7	31.0 ± 0.6	32.2 ± 0.8	19.0 ± 2.1
AI 518	9.9 ± 0.7	9.5 ± 0.6	18.9 ± 1.6	19.6 ± 1.5	13.7 ± 1.1
AI 540	18.3 ± 0.7	18.1 ± 1.3	22.2 ± 2.3	27.4 ± 1.2	18.4 ± 0.7
DMS 4B	13.6 ± 1.4	17.7 ± 0.8	23.1 ± 1.4	21.7 ± 2.0	15.2 ± 1.1
CML 565	26.5 ± 0.6	23.4 ± 0.6	44.6 ± 1.6	34.5 ± 0.4	24.6 ± 2.3
PDM 77-A	19.2 ± 0.9	18.6 ± 2.2	23.1 ± 1.4	19.9 ± 2.4	15.1 ± 2.4
PDIM 639	19.6 ± 2.1	22.0 ± 1.2	23.1 ± 1.6	22.8 ± 1.3	17.2 ± 1.4
PDM 6555	23.2 ± 1.0	17.3 ± 0.9	25.9 ± 1.2	23.0 ± 1.4	14.8 ± 0.6
C 70	23.5 ± 2.1	21.1 ± 0.8	23.3 ± 1.7	21.6 ± 1.0	19.1 ± 2.1
DDM 2309-O	15.1 ± 2.1	20.4 ± 0.7	22.1 ± 0.6	20.7 ± 2.2	19.2 ± 1.1
C 11	22.3 ± 1.2	16.0 ± 2.3	25.0 ± 2.9	24.7 ± 1.6	19.0 ± 0.3
R-lines					
AI 117	16.9 ± 1.0	20.4 ± 1.4	23.1 ± 1.6	24.7 ± 1.5	18.1 ± 1.5
AI 125	28.6 ± 2.1	24.3 ± 1.0	36.0 ± 0.3	36.3 ± 1.3	20.8 ± 1.1
AI 541	13.7 ± 1.4	13.0 ± 1.3	21.3 ± 1.7	18.2 ± 0.2	11.1 ± 0.9
AI 155	15.2 ± 0.4	20.4 ± 2.2	23.9 ± 1.2	22.0 ± 0.9	15.4 ± 0.7
AI 545	18.9 ± 1.9	13.4 ± 0.4	22.5 ± 0.8	15.3 ± 1.0	11.5 ± 1.5
AI 1100	26.5 ± 1.3	22.7 ± 1.4	31.8 ± 1.0	33.3 ± 1.0	21.7 ± 1.4
AI 542	21.2 ± 0.7	33.3 ± 2.0	31.0 ± 0.7	28.3 ± 0.5	24.1 ± 3.1
PML 105	21.2 ± 1.8	18.0 ± 2.0	23.9 ± 0.4	20.1 ± 1.8	19.9 ± 1.1
AI 525	28.0 ± 1.7	19.7 ± 0.5	21.4 ± 0.8	22.0 ± 1.2	16.5 ± 1.5
PDM 24-1	20.1 ± 1.1	18.6 ± 1.5	20.6 ± 1.3	21.4 ± 0.6	14.5 ± 0.5
PDM 4061	14.6 ± 0.2	13.0 ± 0.3	22.2 ± 1.3	23.3 ± 1.9	17.9 ± 1.9
CML 442 (R)	17.5 ± 0.7	18.7 ± 0.6	30.6 ± 2.1	25.6 ± 1.4	20.4 ± 1.0
UMI 1210 (S)	11.7 ± 0.9	9.9 ± 0.2	19.1 ± 1.0	17.9 ± 1.6	11.7 ± 0.7
F-probability	<0.001	<0.001	<0.001	<0.001	<0.001
LSD (*P* = 0.05)	3.88	3.90	4.22	3.94	4.58

The values in the table represent Mean ± Standard error. LSD, Least significant differences; R, Resistant check; S, Susceptible check.

### 3.5 Association of induced biochemical constituents in maize lines with life table parameters of *S. frugiperda*

The r, λ, R_0_ and GRR of *S. frugiperda* developed on diverse maize lines were correlated significantly and negatively; while T and DT were correlated significantly and positively with induced levels of total tannins, ferric ion-reducing antioxidant power (FRAP), ascorbate peroxidase (APX), catalase (CAT), tyrosine ammonia lyase (TAL) and phenylalanine ammonia lyase (PAL), except in few cases (**Table 4**). In contrast, λ, R_0_ and GRR were significantly and positively correlated with total proteins (**Table 4**). However, induced levels of total sugars, phenols, antioxidants and AO in diverse maize lines had no or little effect on life table parameters of S. *frugiperda* (**Table 4**).

**Table 4:** Association of biochemical changes in maize parental lines with various life table parameters of *Spodoptera frugiperda*.

Biochemical constituents (%)	Intrinsic rate of increase	Finite rate of increase	Net reproductive rate	Gross reproduction rate	Mean generation time	Doubling time
Total sugars (X_1_)	0.16	0.36*	0.13	0.306	-0.30	-0.20
Total protein (X_2_)	0.31	0.47**	0.43*	0.53**	-0.19	-0.29
Total phenols (X_3_)	-0.22	-0.36*	-0.27	-0.33	0.25	0.287
Total tannin (X_4_)	-0.47**	-0.45*	-0.43*	-0.48**	0.38*	0.46**
Total antioxidants (X_5_)	-0.33	-0.28	-0.29	-0.22	0.23	0.16
FRAP (X_6_)	-0.37*	-0.54**	-0.48**	-0.55**	0.37*	0.40*
Ascorbate oxidase (X_7_)	-0.29	-0.46**	-0.20	-0.33	0.26	0.190
Ascorbate peroxidase (X_8_)	-0.41*	-0.39*	-0.41*	-0.50**	0.46*	0.38*
Catalase (X_9_)	-0.50**	-0.77**	-0.60**	-0.58**	0.58**	0.58**
Phenylalanine ammonia lyase (X_10_)	-0.39*	-0.57**	-0.49**	-0.50**	0.45*	0.39*
Tyrosine ammonia lyase (X_11_)	-0.48**	-0.43*	-0.43*	-0.46*	0.41*	0.46**

*, ** = Correlation coefficients significant at *P* = 0.05, 0.001, respectively. FRAP, Ferric ion-reducing antioxidant power.

Multiple linear regression

Intrinsic rate of increase = 0.192 - 0.000 X_1_ - 0.000 X_2_ + 0.001 X_3_ - 0.002 X_4_ - 0.000 X_5_ - 0.001 X_6_ + 0.001 X_7_ - 0.001 X_8_ - 0.001 X_9_ + 0.002 X_10_ - 0.001 X_11_ (R^2^ = 53.90)

Finite rate of increase = 1.219 + 0.000 X_1_ + 0.000 X_2_ + 0.001 X_3_ -0.000 X_4_ + 0.002 X_5_ -0.001 X_6_ -0.000 X_7_ + 0.001 X_8_ - 0.007 X_9_ + 0.002 X_10_ + 0.000 X_11_ (R^2^ = 72.80)

Net reproductive rate = 159.366 - 0.733 X_1_ + 0.008 X_2_ + 0.729 X_3_ - 1.588 X_4_ + 0.321 X_5_ -1.308 X_6_ + 1.623 X_7_ -1.150 X_8_ - 2.326 X_9_ + 1.450 X_10_ - 0.185 X_11_ (R^2^ = 65.20)

Gross reproduction rate = 225.602 - 0.512 X_1_ + 0.578 X_2_ + 1.151 X_3_ - 2.885 X_4_ + 2.125 X_5_ - 1.913 X_6_ + 1.938 X_7_ -2.266 X_8_ - 3.200 X_9_ + 1.875 X_10_ + 0.190 X_11_ (R^2^ = 60.30)

Mean generation time = 26.830 + 0.008 X_1_ + 0.046 X_2_ - 0.080 X_3_ + 0.057 X_4_ - 0.089 X_5_ - 0.007 X_6_ - 0.098 X_7_ + 0.244 X_8_ + 0.298 X_9_ - 0.001 X_10_ - 0.012 X_11_ (R^2^ = 57.10)

Doubling time = 2.918 + 0.016 X_1_ + 0.009 X_2_ -0.027 X_3_ + 0.073 X_4_ - 0.031 X_5_ + 0.040 X_6_ - 0.043 X_7_ + 0.028 X_8_ + 0.094 X_9_ -0.075 X_10_ + 0.028 X_11_ (R^2^ = 67.20)

Stepwise regression

Intrinsic rate of increase (per day) = 0.15 - 0.001 X_9_ (R^2^ = 25.00)

Finite rate of increase (per day) = 1.23 + 0.0025 X_5_ - 0.0068 X_9_ (R^2^ = 68.00)

Net reproductive rate (offspring/female) = 117.5 - 1.74 X_9_ (R^2^ = 36.00)

Gross reproduction rate (offspring/female) = 242.34 - 3.31 X_9_ (R^2^ = 34.00)

Mean generation time (days) = 30.54 + 0.18 X_9_ (R^2^ = 34.00)

Doubling time (days) = 4.52 - 0.028 X_3_ + 0.079 X_4_ + 0.037 X_6_ - 0.05 X_7_ + 0.096 X_9_ - 0.096 X_10_ (R^2^ = 58.00)

The multiple linear regression model showed that the per cent increase in the levels of biochemicals in test maize lines have accounted for 53.90, 72.80, 65.20, 60.30, 57.10 and 67.20% variability of the r, λ, R_0_, GRR, T and DT of *S. frugiperda*, respectively (**Table 4**). However, the stepwise regression revealed differences in the contribution of biochemical constituents of maize lines to these growth parameters. Per cent increase of catalase alone have accounted for 25.00, 36.00, 34.00 and 34.00% variability of r, R_0_, GRR and T, respectively. Further, per cent increase of total antioxidants and catalase together have accounted for 68.00% variability in λ. Moreover, per cent change in total phenols, total tannin, FRAP, AO, catalase and PAL together have contributed 58.00% variability to DT of *S. frugiperda* (**Table 4**).

## 4 Discussion

The life cycle of herbivorous insects and their population dynamics are closely interconnected and significantly affected by variations in nutrient contents among different host plants (Sedighi et al., 2017; Wu et al., 2019; Ribeiro et al., 2020; He et al., 2021b; Chen et al., 2022). When an insect consumes a particular host plant and shows increased developmental time along with decreased reproduction rates compared to other host plants, that plant is regarded as resistant (Chen et al., 2018). The biological parameters R₀, r, λ, T and *v_xj_* are the primary determinants of variations in growth, development, reproduction, and survival of insects, providing valuable insights into their potential for population growth in specific environments (Wu et al., 2006). The *S. frugiperda* fed on A-lines CML 565, PDIM 639, DDM 2309-O, AI 196 and AI 544, and R-lines AI 125, AI 155, AI 1100 and PML 105 had lower r, λ and R₀ as compared to other lines. Among them, *v_xj_* was very much lower on CML 565, AI 544, AI 125 and AI 155 compared to resistant check CML 442. Conversely, T and DT of *S. frugiperda* on these lines were higher than other lines suggesting these lines exhibited resistant reaction against *S. frugiperda*. Zhang et al. (2021) reported that feeding preference of *S. frugiperda* larvae for distinct types of maize was varied according to developmental stage and feeding period. Further, Zhang et al. (2023) observed that, the resistant maize varieties showed lower r, λ, Ro and GRR and longer T of *S. frugiperda*. Some parental maize lines have resulted in higher r, λ and T of *S. frugiperda* indicating susceptible reaction in those lines and making them suitable hosts (Hong et al., 2022). Similar findings were also reported where *S. frugiperda* had longer developmental duration on resistant variety than on the susceptible one (Ng et al., 1985; Chang et al., 2000; Nelly et al., 2023). Plant species have an impact on the pupal weight of the fall armyworm (Wang et al., 2020) and various food plants influence the reproductive capacity of insects (Pencoe and Martin, 1981). The accumulation of nutrition during the immature and adult stages is the primary determinant of adult fertility (He et al., 2021a). Additionally, the pupal mass and reproductive output of female lepidopterans were positively correlated with their adaptability potential (Takahashi et al., 2012).

The chemical composition of the host plant can be altered during stress conditions (Kumar, 1997; Chiriboga Morales et al., 2021) and which can influence insect performance in either a positive (Wu et al., 2021); or negative way (Acharya et al., 2022). Among secondary metabolites, phenols are particularly significant for their role in combating insect pests (Sharma et al., 2009). Oxidation of phenolic compounds result in production of toxic quinones which affect the insect growth and development, however some phenols are directly toxic to insect pests (Howe and Jander, 2008; Naoumkina et al., 2010; Vashisth et al., 2022). Tannins, along with oxidative enzymes and proteinase inhibitors, are known to be systemically activated in leaves following herbivore-induced injury (Peters and Constabel, 2002). In maize, the chemical composition of the foliage directly influences the feeding behavior of *S. frugiperda* (Zhang et al., 2021). In response to such herbivory, plants enhance their natural defenses by producing secondary metabolites and antioxidative enzymes (Bhoi et al., 2020; Jan et al., 2021). To better understand these insect-plant interactions, it is essential to analyze defensive phytochemicals in test plants that could influence the biology of *S. frugiperda* (Hong et al., 2022). Our findings, revealed that nutritional (sugars and proteins), secondary metabolites (tannins and phenols), total antioxidants and FRAP increased significantly in infested maize lines, particularly in A-lines CML 565, AI 501, AI 1116, DMS 4B, and R-lines AI 125, AI 1100 and AI 542, compared to healthy plants. The responses intensity varied among maize lines, indicating that some lines possess stronger defense mechanisms against insect attack than others. These results align with earlier studies by Cabezas et al. (2013) and da Silva et al. (2017) who reported that the nutritional level significantly influences the developmental parameters of *Spodoptera sp* and can vary among different host plants. Comparable results were observed in resistant germplasm lines which impede larval growth and prolong the pre-pupal and pupal stages of *S*. *frugiperda* after herbivory (Chen et al., 2009; Yang et al., 2023). Further supporting our findings, studies by Bhoi et al. (2020) and Sau and Dhillon (2022) demonstrated that resistant maize genotypes showed a higher percentage increase in secondary metabolites, total antioxidants and FRAP levels compared to susceptible genotypes in response to damage by *Chilo partellus* and *Sesamia inferens*, respectively. Furthermore, Dhillon and Chaudhary (2015, 2018) also highlighted that maize and sorghum genotypes with varying resistance or susceptibility exhibit distinct nutritional profiles, influencing their interactions with *C. partellus*.

Following insect feeding in maize, the plants activated various oxidative enzymes (Usha Rani and Jyothsna, 2009; War et al., 2011, 2012; Bhoi et al., 2020). Antioxidant enzymes are well-known for being efficient ROS scavengers and for being essential for regulation of levels of ROS (Nadarajah, 2020). Their activation is crucial, as these enzymes play a vital role in the plants defense against pest attack. In the current study there is a significant upsurge of AO, APX, catalase PAL, and TAL enzymes across various test maize lines in *S. frugiperda* damage conditions. However, the percentage increase in these enzymes under *S. frugiperda* damage varied among the maize genotypes for different enzymes. Earlier studies also have reported enhanced PAL activity in plants following insect damage (Zhao et al., 2009; Chen et al., 2009). Similar findings were reported by Costa et al. (2020), who observed increased levels of superoxide dismutase (SOD) and peroxidase (POD) in cowpea plants with *S. frugiperda* damage, is an indicative of higher resistance levels. Similarly, Kumar et al. (2023) reported that antioxidant enzymes like AO, APX, catalase, PAL and TAL increased in the seedlings of tested sorghum genotypes as compared to susceptible under *C. partellus* damaged conditions.

The association between *S. frugiperda* life table parameters and induced biochemicals in test maize parental lines demonstrated that the secondary metabolites and antioxidant enzymes had significantly negative correlation with r, λ, R_0_ and GRR. Conversely, these metabolites and enzymes had a significant and positive association with T and DT of *S. frugiperda*. Felton and Summers (1993), reported similar findings, noting that the production of APX in soyabean leaves negatively impacted the growth and development of *Helicoverpa zea* caterpillar. These association studies between life tale characteristics of *S. frugiperda* and the induced biochemicals in maize parental lines have clearly demonstrated the antibiosis effect of resistant maize parental lines. Specifically, maize lines with higher levels of defensive biochemicals showed to prolong the developmental durations of *S. frugiperda* while simultaneously reducing its fecundity.

## 5 Conclusion

The A-lines CML 565, AI 501, AI 544 and PDIM 639, and R-lines AI 125, AI 542, AI 155, AI 1100 and PML 105 were found to impart detrimental effect on various life table parameters of *S. frugiperda*, and had greater insect-induced increase in the test phytochemicals and defense enzymes in comparison to other test maize lines. The findings highlight the significance of the life table analysis and biochemical profiling in identifying resistant maize parental lines against *S. frugiperda*. These parental inbred lines could be used in the hybrid breeding program to develop *S. frugiperda* resistant maize hybrids.

## Data Availability

The raw data supporting the conclusions of this article will be made available by the authors, without undue reservation.
